# Warming impact on herbivore population composition affects top-down control by predators

**DOI:** 10.1038/s41598-017-01155-y

**Published:** 2017-04-19

**Authors:** Ying-Jie Wang, Takefumi Nakazawa, Chuan-Kai Ho

**Affiliations:** 1 Institute of Ecology and Evolutionary Biology, National Taiwan University, Taipei, Taiwan; 2 Department of Life Sciences, National Cheng Kung University, Tainan, Taiwan; 3 Department of Life Science, National Taiwan University, Taipei, Taiwan

## Abstract

Understanding warming impact on herbivores facilitates predicting plant/crop dynamics in natural/agricultural systems. However, it remains unclear how warming will affect herbivore population size and population composition, consequently altering herbivore colonization in a tri-trophic system (plant-herbivore-predator or crop-pest-biocontrol agent). We studied a soybean-aphid-lady beetle system, by conducting (1) a laboratory warming experiment to examine warming impact (+2 °C or +4 °C) on the aphid population size and composition (alate proportion), and (2) a field colonization experiment to examine whether the warming-induced effect subsequently interacts with predators (lady beetles) in affecting aphid colonization. The results showed that warming affected the initial aphid population composition (reduced alate proportion) but not population size; this warming-induced effect strengthened the top-down control by lady beetles and slowing aphid colonization. In other words, biocontrol on crop pests by predators could improve under 2–4 °C warming. Furthermore, aphid colonization was affected by an interaction between the alate proportion and predator (lady beetle) presence. This study suggests that warming affects herbivore population composition and likely mediates top-down control on herbivore colonization by predators. This mechanism may be crucial but underappreciated in climate change ecology because population composition (wing form, sex ratio, age/body size structure) shifts in many species under environmental change.

## Introduction

Herbivores exert top-down control on plant growth, development, and production in natural and agricultural systems^1–3^. Climate warming has the potential to affect this top-down control by herbivores. This could shape wild plant dynamics and crop production, which are associated with ecosystem stability and food security, respectively. Warming impact on herbivore populations may benefit or damage plants. For example, in agroecosystems, a positive population response of herbivores (crop pests) to warming can damage plant (crop) performance and production^4, 5^. By contrast, a negative population response of herbivores to warming can benefit plants because under warming, herbivores develop and reproduce suboptimally^6^, or the top-down control of herbivores by predators is stronger^7^.

Although studies have highlighted the important role of herbivore dynamics in evaluating climate warming impact on natural or agricultural systems, some critical knowledge gaps remain. For example, many studies have focused on warming impact on herbivore population size^8–10^, and less is known about (1) whether warming affects not only population size but also population composition in herbivores (e.g., the ratio of alate to apterous forms - morphological traits related to herbivore migration ability), and (2) how this warming impact may subsequently affect herbivore (pest) colonization on plants (crop) in the presence or absence of predators (biocontrol agent). Given that average global surface temperature may increase from 1.9 to 3.7 °C by 2100 (RCP 4.5 and 8.5)^11^, is it possible that this degree of warming will be just sufficient to change one but not another herbivore population trait (e.g., change in population composition, but not population size)? In addition, will this warming impact on herbivore population subsequently interact with predator effect in influencing herbivore colonization (pest outbreak)? Answering these questions can advance our understanding in basic ecology (i.e., warming impact on a tri-trophic system), as well as applied ecology (i.e., warming impact on crop pest outbreak and biocontrol effectiveness).

To understand the mechanisms underlying warming impact, this study examined a soybean-aphid-lady beetle system to answer the following questions: (1) *How would 2–4* °C *warming affect the population size and composition (alate vs. apterous) of aphids?* (2) *How would this warming impact on the initial population size and composition subsequently interact with lady beetle (predator) effect in influencing aphid colonization?* Previous studies hint that warming may alter the aphid population composition by directly reducing or indirectly increasing alates (winged aphids) through a crowding effect (increased population size)^12–14^. Because alates have a better migration ability^15, 16^, warming impact on the aphid population composition (alate proportion) likely affects subsequent aphid colonization.

**Figure 1:**
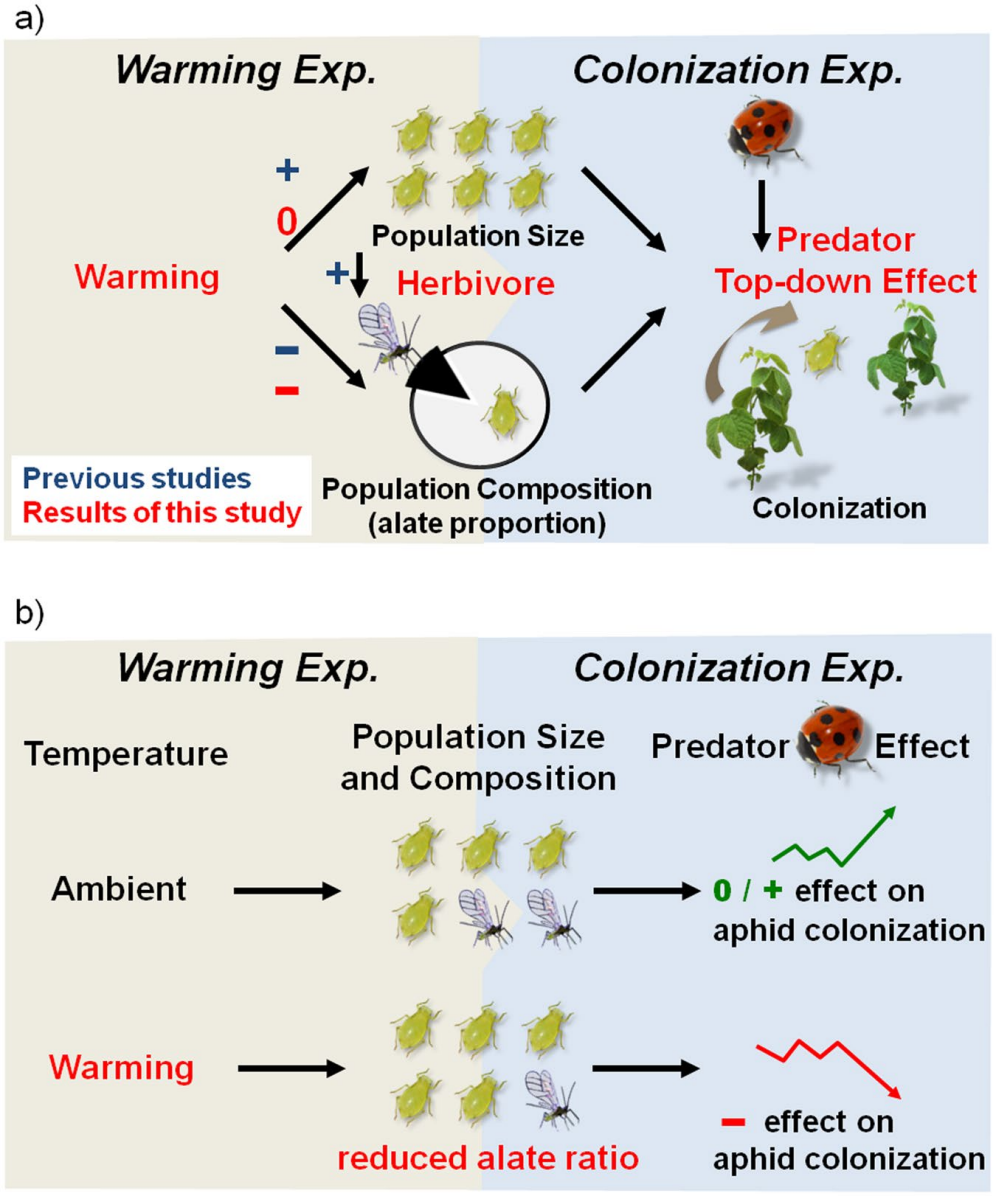
(**a**) The concept map of this study, including two experiments: *warming experiment* examining warming impact on herbivore population size and composition, and *colonization experiment* examining the subsequent effect on herbivore colonization with/without predators. While previous studies suggested that higher aphid population size could lead to higher alate proportion (crowding effect), this study did not explore this relationship because the *warming experiment* showed no warming impact on aphid population size. Note that +, −, and 0 indicate positive, negative, and no effects, respectively. (**b**) The simplified results of this study: warming affected the herbivore population composition (reduced alate proportion) without changing the population size, and this warming-induced effect could interact with predator (lady beetle) effect in influencing herbivore colonization. Note that +, −, and 0 indicate the positive, negative, and no effects, respectively, of predator presence on aphid colonization.

This study included both laboratory and field experiments (warming and colonization experiments, respectively; Fig. 1a), focusing on soybean (*Glycine max*), soybean aphids (*Aphis glycines*), and seven-spotted lady beetles (*Coccinella septempunctata*) because they represent a typical plant-herbivore-predator system, as well as an important crop, crop pest and biocontrol agent, respectively. We first conducted a warming experiment in growth chambers to examine how the predicted warming of 2–4 °C by 2100^11^ will affect the population size and composition (alate proportion) of soybean aphids. The ambient (control) temperature was set at 24.5 °C, reflecting the average temperature of the soybean growth season and nearing the optimum growth temperature of soybean aphids (Supplement 1). The results showed that warming suppressed alate production from approximately 5% to 1% but did not affect the overall population size of aphids (Fig. 1b). On the basis of these results, a colonization experiment (without active warming) was then conducted in the field with a 2 (5% vs. 1% alate proportion representing ambient vs. warming scenario) × 2 (lady beetle presence vs. absence) factorial design to investigate how warming-induced change in the aphid population composition (reduced alate proportion) may affect aphid colonization on soybean plants with/without lady beetles. The results showed that temperature impact on the aphid population composition interacted with lady beetle treatment in affecting subsequent aphid colonization (Fig. 1b). For instances, a warming-induced reduction in alate aphids could increase the biocontrol effectiveness of lady beetles (e.g., inhibiting aphid colonization), suggesting that the value of biocontrol increases under warming.

## Results

### Warming experiment

**Figure 2:**
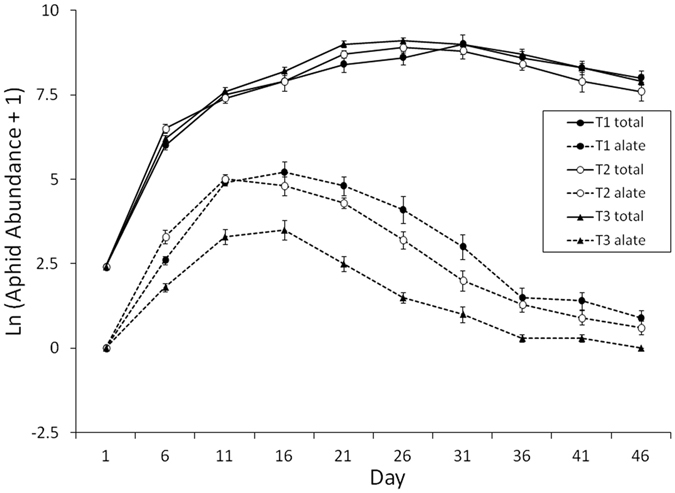
Temperature effect on the total aphid population size (solid lines; total = alate + apterous) and alate population size (dashed lines) on a soybean plant over time (Mean ± SE; T1 = 24.5 °C, T2 = 26.5 °C, T3 = 28.5 °C).

Warming by 2 or 4 °C altered the aphid population composition but not the overall population size. Specifically, warming reduced alate production (*P* = 0.013; Table 1 and Fig. 2), but did not affect the aphid population size (*P* = 0.636; Table 1 and Fig. 2). Under ambient, 2, and 4 °C warming treatments, the average percentages of alates in the aphid population were 5.51, 5.01, and 1.34%, respectively (Supplement 4).

**Table 1:** Statistical results in the warming experiment: effects of temperature treatment and time on aphid and alate abundance.

DenomDF = 284	NumDF	Aphid abundance		Alate abundance	
Variable		*F*	*P*	*F*	*P*
Temperature (T)	2	0.45	0.636	4.41	**0.013**
Time (in days) (D)	1	15.60	**<0.001**	4.34	**0.038**
T × D	2	0.21	0.811	0.55	0.576

The statistical analysis applied linear model using generalized least squares (GLS) with AR1 correlation structure to account for repeated measures of each aphid population. Time effect was treated as a continuous (numeric) variable due to a linear relationship between time and aphid population size.

### Colonization experiment

**Figure 3:**
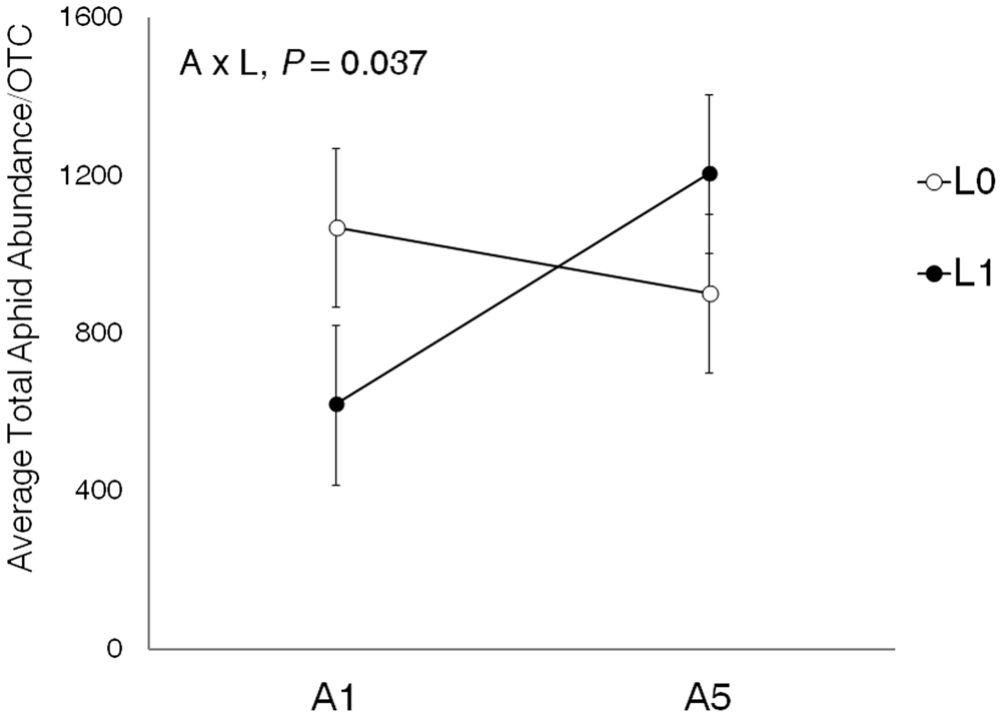
Alate proportion treatment (A) and lady beetle treatment (L) interacted and affected aphid populations in the colonization experiment (statistical results reported in Table 2). A1 = low alate proportion (1%, warming scenario); A5 = high alate proportion (5%, ambient-temperature scenario); L0 = lady beetle absence; L1 = lady beetle presence. Each point represents the average total aphid abundance per OTC (least square means ± SE) over the study period.

**Figure 4:**
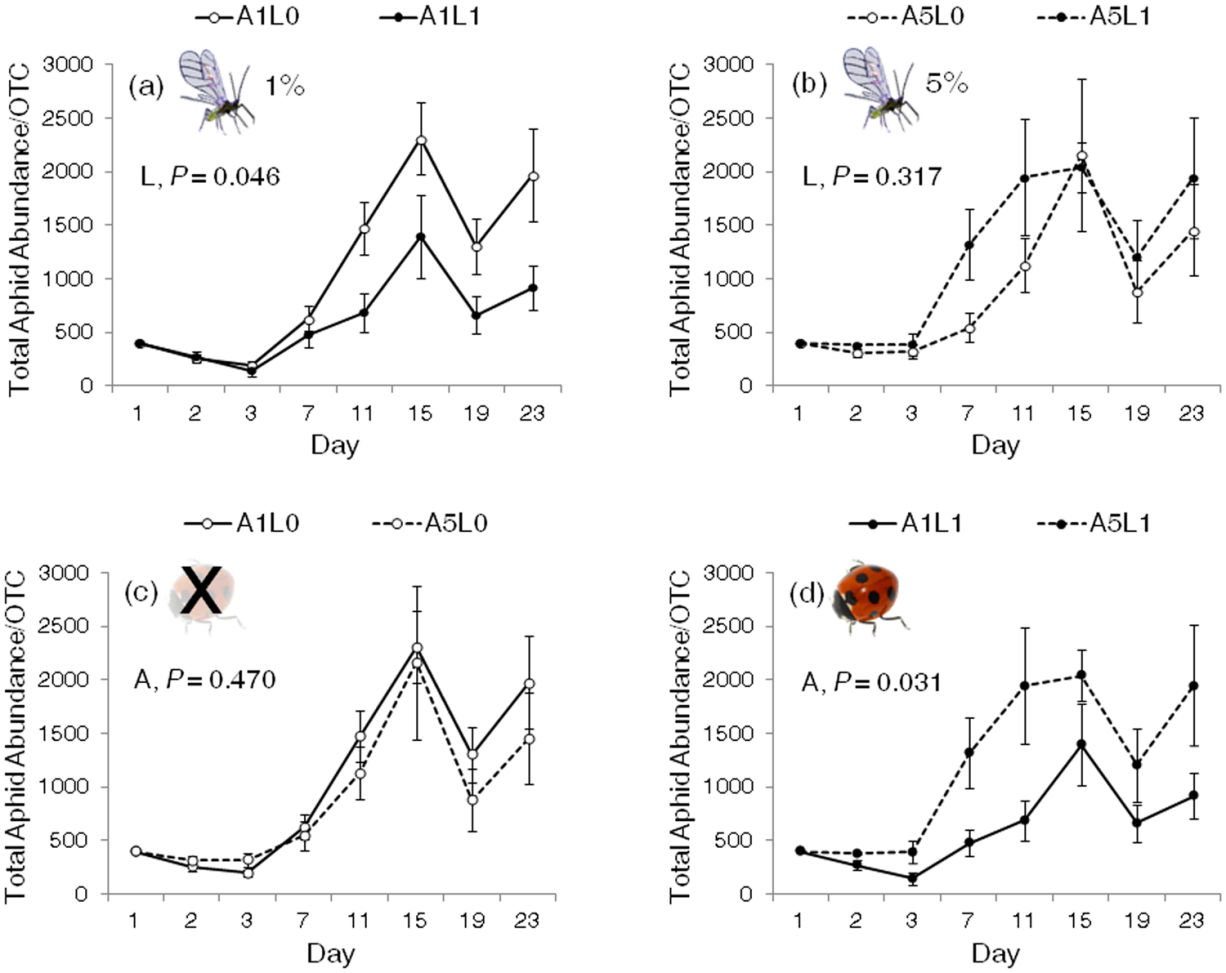
Aphid abundance on all soybean plants in an OTC under a combination of alate proportion treatment (A) and lady beetle treatment (L): A1 = low alate proportion (1%, warming scenario); A5 = high alate proportion (5%, ambient-temperature scenario); L0 = lady beetle absence; L1 = lady beetle presence. To facilitate the comparison of results, four sub-figures are listed: (**a**) A1L0 vs. A1L1, (**b**) A5L0 vs. A5L1, (**c**) A1L0 vs. A5L0, and (**d**) A1L1 vs. A5L1. When alate proportion was low (1%; warming scenario), lady beetle presence reduced aphid abundance (*P* = 0.046; Fig. 4a). When alate proportion was higher (5%; ambient scenario), this pattern was not observed (Fig. 4b), while lady beetle presence facilitated aphid population growth on Day7 (*P* = 0.0131). A different way to examine the interaction between alate proportion and lady beetle treatments are shown in Fig. 4c and d: lower alate proportion treatment impeded aphid colonization only when lady beetles were present (*P* = 0.031; Fig. 4d). Each point represents Mean ± SE.

The interaction between alate proportion treatment (ambient vs. warming scenario) and lady beetle treatment affected aphid abundance in the OTCs (*P* = 0.037; Table 2 and Fig. 3). Under lower alate proportion treatment (1%, warming scenario), aphids colonized all soybean plants at a lower density in the presence than in the absence of lady beetles (Fig. 4a), suggesting effective top-down control (biocontrol) on aphid colonization by lady beetles across all soybean plants (central and neighbor plants; definition in Supplement 3). However, under higher alate proportion treatment (5%, ambient-temperature scenario), this top-down control on aphids was not observed across all soybean plants (Fig. 4b). Unexpectedly, aphids colonized faster in the presence than in the absence of lady beetles on Day 7 (*P* = 0.0131, LSMeans) (Fig. 4b), suggesting that under a higher alate proportion, lady beetles’ presence could “promote” aphid colonization across all soybean plants. Furthermore, under lady beetle absence treatment, alate proportion treatment did not affect aphid colonization (Fig. 4c). However, under lady beetle presence treatment, a higher alate proportion facilitated aphid colonization (Fig. 4d).

**Table 2:** Statistical results ﻿in the colonization experiment: effects of alate proportion treatment, lady beetle treatment, and time on aphid abundance in an OTC, which included one central and four neighbor plants.

DenomDF = 152	Aphid Abundance in an OTC (A/OTC)		
Variable	NumDF	*F*	*P*
Alate proportion (A)	1	1.30	0.256
Lady beetle (L)	1	0.32	0.573
A × L	1	4.45	**0.037**
Time (in days) (D)	1	44.26	**<0.001**
A × D	1	0.18	0.672
L × D	1	0.44	0.506
A × L × D	1	1.46	0.229

The statistical analysis applied linear model using generalized least squares (GLS) with AR1 correlation structure to account for repeated measures of each aphid population. Time effect was treated as a continuous (numeric) variable due to a linear relationship between time and aphid population size. Box-Cox transformation was applied to improve normality.

To further investigate aphid colonization, we examined the aforementioned aphid abundance on central and neighbor plants separately (Supplement 5). The patterns of aphid abundance on the central and neighbor plants were similar to that on all soybean plants (Supplement 6-1 and 6-2 for the central and neighbor plants, respectively). We also analyzed alate aphid abundance on all, central, and neighbor plants and observed less consistent results (Supplement 7-1, 7-2, and 7-3, respectively), likely due to the high mobility of alate aphids.

## Discussion

The top-down control of plants by herbivores is critical for determining wild plant dynamics and crop production (food security). To understand how the herbivore impacts will change under climate warming, many studies have focused on the direct and indirect effects of warming on herbivore population size. This study, however, highlights the need to also consider warming impact on herbivore population composition, demonstrating that (1) warming could affect herbivore population composition (i.e., reduced alate proportion) without changing population size, (2) this warming-induced effect on population composition subsequently inhibited herbivore colonization under predator presence, suggesting higher biocontrol effectiveness under warming, and (3) herbivore population composition interacted with predator effect in influencing herbivore colonization.

### Direct effects of warming on population size

The results of our warming experiment demonstrated no direct effects of warming on herbivore (aphid) population size, in contrast to previous studies where a direct, positive effect of warming on herbivore population size was found (Fig. 1a)^17–19^. This difference may be because our warming temperatures occurred within the optimum growth temperature range of herbivores (Supplement 8). If both ambient (control) and elevated temperature (warming) treatments are located in the plateau of the thermal performance curve (i.e., optimum temperature), the degree of warming may not appreciably change herbivore population size.

### Direct and indirect effects of warming on population composition

Warming is predicted to exert conflicting effects on alate production–a positive indirect effect (crowding effect) or a negative direct effect (Fig. 1a)^12, 13^. However, in this study, warming had a negative direct effect on the alate proportion alone, without increasing the aphid population size (crowding effect), which could have generated the positive indirect effect. While the negative direct effect of warming outweighed the positive indirect effect, their relative strength may change outside the optimum temperature range of soybean aphids; for example, the indirect effect may be stronger below the optimum temperature range (Lin and Ho, *unpublished data*).

### Populations size vs. population composition

Although warming by 4 °C did not affect the initial aphid population size, it was sufficient to alter the population composition (reduced alate proportion). This warming-induced effect (i.e., alate reduction) subsequently inhibited aphid colonization in the tri-trophic system (Figs 1b and 4d). The results have two major implications: (1) Although we might not observe a direct effect of climate warming on the initial population size of a species, warming may still indirectly affect the subsequent population size by altering the population composition. (2) To better predict climate warming impact, future studies should examine warming effects on not only population size but also population composition, such as wing type (alate vs. apterous), sex ratio, age structure, and body size variation, all of which could critically affect species colonization.

### Interaction between alate proportion and lady beetle presence

The colonization experiment revealed an interaction between alate proportion and lady beetle treatments (Table 2; Figs 1b, 3 and 4). When alate proportion was low (1%; warming scenario), lady beetles’ presence reduced aphid colonization (Fig. 4a), suggesting that biocontrol effectiveness may increase with warming. However, when alate proportion was higher (5%; ambient scenario), lady beetles’ presence did not suppress aphid populations (Figs 3 and 4b) or could even facilitate aphid colonization (Day 7 in Fig. 4b), likely explaining the failure of some biocontrol practices. This interaction suggests that the impact of lady beetles on aphid colonization depends on alate proportion (warming vs. ambient scenario). When alate proportion is low, classic top-down control of aphids by lady beetles occurs (Fig. 4a). When alate proportion is higher, more alate aphids may increase the randomness of aphid distribution, reduce predator efficiency, and benefit the overall aphid population (Fig. 4b)^20^. In addition, the less aggregated and escaped aphids may increase their reproduction because of a smaller crowding effect (colonizing new plants under predator disturbance) or a compensatory response to predation risk (e.g., alarm pheromone effect^21^). This may explain why we observed a larger aphid population size in the presence of lady beetles in the higher alate treatment, as shown for aphid populations on all plants (Day 7 in Fig. 4b). Although not explicitly tested, our aforementioned hypothesized mechanisms could theoretically produce results consistent with our field results (Supplement 9).

### Potential caveats of this study

The warming experiment was conducted under constant temperature due to logistics. Therefore, the results does not reflect how temperature fluctuation (e.g., hourly) may affect aphid population size and composition. In addition, the field colonization experiment involved applying the warming experiment results (i.e., lower alate proportion under warming) to assess warming impact on aphid colonization, instead of applying real temperature manipulation. This approach does not account for potential warming impact on plants and lady beetles (and then on aphids), but this should not compromise our conclusions because of these reasons. First, warming treatment up to 4 °C, if included in the colonization experiment, should not change plant nutritional and defensive quality (bottom-up control of aphids) (Supplement 10). Second, the warming treatment will likely increase lady beetles’ predation efficiency on aphids, thereby strengthening our conclusion that warming-induced effect on aphid population composition could increase the top-down control of aphids by lady beetles (Supplement 10).

### Conclusions

This study implies that warming around the optimum temperature of soybean aphids (crop pest) may not increase the initial aphid population size but may limit their colonization potential, by changing the population composition (reduced alate proportion) and then likely increasing aphid susceptibility to top-down control by lady beetles. In other words, biocontrol using predators may be more effective for limiting pest outbreaks and protecting crops under future climate warming in this or similar systems. Furthermore, many species respond to environmental change by shifting their population composition, such as the alate or macropterous proportion (aphids, planthoppers), sex ratio (reptiles, birds), age structure (fish, amphibians), and body size structure (fish, insects), all of which could critically affect species colonization. Therefore, understanding warming impact on population composition and its subsequent effect on population dynamics is an important, but underappreciated, topic in climate change ecology.

## Methods

### Species

This study focused on soybean (*G. max* (L.) Merrill), soybean aphids (*A. glycines* Matsumura), and seven-spotted lady beetles (*C. septempunctata* Linnaeus). Soybean is one of the most important crops worldwide. Soybean aphids, which originated from Asia, have invaded North America and reduced soybean production. Seven-spotted lady beetles are commonly used worldwide as a biocontrol agent to suppress aphid population (more information in Supplement 1). This study used a commercial cultivar of soybeans (*G. max* cv. Kaohsiung No. 9) and collected soybean aphids and lady beetles from multiple sites to avoid idiosyncratic effect (more information on stock establishment in Supplement 2).

### Warming experiment

To test warming impact on aphid population size and composition (apterous vs. alate), we set up aphid colonies at 24.5, 26.5, and 28.5 °C, representing ambient, 2 °C, and 4 °C warming treatments, respectively. The temperature settings were based on (1) the mean temperature of soybean season (24.7 °C) in Hualien, Tainan, Kaohsiung, Hengchun, and Taipei, Taiwan from 1981 to 2010, (2) the degree of warming predicted by the end of this century (IPCC 2014), and (3) the optimal thermal range of soybean aphids (25–30 °C, Supplement 1). Each treatment had 10 replicates, each of which initially included 10 apterous (wingless) aphids on a caged, potted soybean plant (V1 stage: first set of true leaves unfolded). The aphid density and infested plant stage were consistent with our field observations. The cage was composed of fine silk meshes (400 holes/cm^2^). The aphid population on each plant was recorded every 5 days for 45 days.

### Colonization experiment

After examining warming impact on aphid population size and composition (i.e., *warming experiment*), we investigated the subsequent effects of warming on aphid colonization in the presence or absence of lady beetles (i.e., *colonization experiment*). Because the warming experiment showed that 2–4 °C warming did not affect aphid population size but reduced alate proportion from approximately 5% to 1%, the colonization experiment was performed using a 2 × 2 factorial design with alate proportion treatment (1% and 5% representing warming and ambient-temperature scenarios, respectively) and lady beetle treatment (presence and absence of lady beetles). The initial population density for aphids (400) and lady beetles (1 or 0) per aphid-infested soybean plant was based on our observations after alates became abundant in the field. This experiment (N = 20) was conducted with open-top chambers (OTCs, Supplement 3) and received no active warming. Each OTC had one aphid-infested plant in the center (central plant), surrounded by four uninfested plants (neighbor plants). Aphid populations on central and neighbor plants were monitored every 24 hrs for the first 3 days and every 4 days thereafter until the 23rd day.

### Data analysis

This study applied a linear model using generalized least squares (GLS) with an AR1 correlation structure (nlme package, R [v.3.2.3]), considering heteroscedasticity and temporal autocorrelation (repeated measures). To examine warming impact on the aphid population size over time (warming experiment), we analyzed the effect of temperature treatment (T) and time (D) by using GLS with AR1 to account for repeated measures of each aphid population. To examine the subsequent effects of warming on aphid population growth and dispersal with or without lady beetles (colonization experiment), we analyzed the effect of alate proportion treatment (A), lady beetle treatment (L) and time (D) by using GLS with AR1 to account for repeated measures of aphid populations in each OTC. When factor interactions (e.g., A × L) were significant, we conducted simple effects tests to examine the significance of one factor at each level of another factor. When applicable, ANOVA and LSMeans tests were performed to assess treatment effects on specific days. To further understand the aphid colonization process, we analyzed the overall aphid population size in an OTC (containing one central and four neighbor plants), as well as the aphid population size on the central plant or four neighbor plants only (Supplement 5).

## Electronic supplementary material

Manuscript Supplementary Information
