# Supplementary material for: Warming impact on herbivore population composition affects top-down control by predators
Source: Sci Rep. 2017 Apr 19;7:941. doi: 10.1038/s41598-017-01155-y (PMC5430442; doi:10.1038/s41598-017-01155-y)
Supplement: Supplementary file 1 — Manuscript Supplementary Information [file 41598_2017_1155_MOESM1_ESM.pdf]

Warming impact on herbivore population composition affects top-down control by predators

Ying-Jie Wang[1], Takefumi Nakazawa[2], Chuan-Kai Ho[1,3*]

[1]Institute of Ecology and Evolutionary Biology, National Taiwan University, Taipei, Taiwan

[2]Department of Life Sciences, National Cheng Kung University, Tainan, Taiwan

[3]Department of Life Science, National Taiwan University, Taipei, Taiwan

* Corresponding author. Email: ckho@ntu.edu.tw

Supplement 1. Focal species in this study.

Soybean, *Glycine max* (L.) Merr. (Fabaceae), an annual herbaceous legume, originates from eastern Asia and has been domesticated from wild soybean (*G. soja* Siebold & Zucc)[1]. It was introduced to Africa in the 1850s, Europe in the 1900s, Australia in the 1910s, and America in the 1930s. Globally, it is one of the most important crops cultivated (whole-bean world trade value at US$53.2 billion in 2012) as human and livestock food and for protein and oil[2,3,4,5,6]. The optimum growth temperature for soybean is 18 – 35°C; fresh beans can be harvested from 45 to 65 days after sowing, and dry beans (14% less water content) can be harvested approximately 100 days after sowing[7]. In this study, the vegetative or green soybean *G. max* cv. Kaohsiung No. 9, created by the Kaohsiung District Agricultural Research and Extension Station (KDAIS; 22°42′33.07″N, 120°31′28.68″E), Taiwan, has become one of Taiwan's most important crops for exportation (grossing more than US$64.4 million/year)[8]. This soybean cultivar is grown by large commercial farms in Southern Taiwan and small farms in Northern and Central Taiwan mostly during early spring (mid-January to mid-March) and then during early fall (mid-August to October). However, this cultivar is not particularly resistant to soybean aphids.

The soybean aphid *Aphis glycines* Matsumura (Homoptera: Aphididae), which

originated from eastern Asia, is heteroecious and holocyclic (host alternating with sexual reproduction during parts of its life cycle). It is considered a secondary insect pest in all soybean-growing regions of China, and it invaded North America around 2000, causing a serious impact on soybean production[9,10,11]. The optimum growth temperature for soybean aphids is 25 – 30°C[12]. In general, up to 10 – 22 generations of soybean aphids can occur during the growth season of soybean plants through parthenogenesis, producing less mobile wingless females (apterae) and highly mobile winged females (alates) that facilitate a wide-range dispersal[13]. As sap suckers, soybean aphids can cause various damages to soybean plants through photosynthate removal, physiological disruption, and virus transmissions[14,15,16,17,18]. The economic loss caused by soybean aphids is estimated to be US$2.4 – 4.9 billion/year[19,20].

The seven-spotted lady beetle (C7), *Coccinella septempunctata* Linnaeus (Coleoptera: Coccinellidae), is a ubiquitous predatory insect originating from Europe and Asia. As an efficient aphidophagous species, it was intentionally introduced to the United States from the 1950s to the 1970s to control the aphid population on crops and has spread across North America since then[21]. The optimum temperature for C7's larval development is 26 – 35°C[22,23]. Their whole-life survival rate, oviposition, and food utilization are greatest at

approximately 25°C [23,24], whereas their intrinsic growth rate peaks at approximately 28°C[25]. In general, they can sexually reproduce 2 – 5 generations/ year[26].

Literature Cited for Supplement 1

1. Kollipara, K. P., Singh, R. J. & Hymowitz, T. Phylogenetic and genomic relationships in the genus *Glycine* Willd. based on sequences from the ITS region of nuclear rDNA. *Genome.* **40**, 57-68 (1997).
2. Shurtleff, W., & Aoyagi, A. *History of soy sauce (160 CE to 2012).* (Soyinfo Center, 2012).
3. Masuda, T., & Goldsmith, P. D. World soybean production: area Harvested, yield, and long-term projections. *Int. Food Agribus. Man.* **12**, 143-162 (2009).
4. Hartman, G. L., West, E. D. & Herman, T. K. Crops that feed the world 2. soybean-worldwide production, use, and constraints caused by pathogens and pests. *Food Secur*. **3**, 5-17 (2011).
5. Ray, D. K., Ramankutty, N., Mueller, N. D., West, P. C. & Foley, J. A. Recent patterns of crop yield growth and stagnation. *Nat. Commun*. **3**, 1293-1299 (2012).
6. Potts, J., Lynch, M., Wilkings, A., Huppé, G., Cunningham, M. & Voora, V. *The State of sustainability initiatives review: standards and the green economy.* (International Institute for Sustainable Development and the International Institute for Environment and Development, 2014.)

7. Steduto, P., Hsiao, T. C., Fereres, E. & Raes, D. Crop yield response to water. *FAO Irrigation and Drainage Paper* No.66 (Food and Agriculture Organization of the United Nations, 2012).

8. Kaohsiung District Agricultural Improvement Station (KDAIS).青年農民台北開國產大豆直營店 要讓國人吃出鮮美與健康. *KDAIS News no.103057* (2014).

9. Liu, J., Wu, K., Hopper, K. R. & Zhao, K. Population dynamics of *Aphis glycines* (Homoptera: Aphididae) and its natural enemies in soybean in northern China. *Ann. Entomol. Soc. Am*. **97**, 235-239 (2004).

10. Ragsdale, D. W., Voegtlin, D. J. & O'Neil, R. J. Soybean aphid biology in North America. *Ann. Entomol. Soc. Am*. **97**, 204-208 (2004).

11. Ragsdale, D. W., Landis, D. A., Brodeur, J., Heimpel, G. E & Desneux, N. Ecology and management of the soybean aphid in North America. *Annu. Rev. Entomol*. **56**, 375-399 (2011).

12. McCornack, B. P., Ragsdale, D. W. & Venette, R. C. Demography of soybean aphid (Homoptera: Aphididae) at summer temperatures. *J. Econ. Entomol*. **97**, 854-861 (2004).

13. Wu, Z., Schenk-Hamlin, D., Zhan, W., Ragsdale, D. W. & Heimpel., G. E. The soybean aphid in China: a historical review. *Ann. Entomol. Soc. Am*. **97**, 209-218 (2004).

14. Myers, S. W., Hogg, D. B, & Wedberg, J. L. Determining the optimal timing of foliar insecticide applications for control of soybean aphid (Hemiptera: Aphididae) on soybean. *J. Econ. Entomol*. **98**, 2006-2012 (2005).

15. Ragsdale, D. W. et al. Economic threshold for soybean aphid (Hemiptera : Aphididae). *J. Econ. Entomol*. **100**, 1258-1267 (2007).

16. Beckendorf, E. A., Catangui, M. A. & Riedell, W. E. Soybean aphid feeding injury and soybean yield, yield components, and seed composition. *Agron. J*. **100**, 237-246 (2008).

17. Hill, J. H., Alleman, R., Hogg, D. B. & Grau, C. R. First report of transmission of soybean mosaic virus and alfalfa mosaic virus by *Aphis glycines* in the New World. *Plant Dis*. **85**, 561 (2001).

18. Wu, Z., Schenk-Hamlin, D., Zhan, W., Ragsdale, D. W. & Heimpel, G. E. The soybean aphid in China: a historical review. *Ann. Entomol. Soc. Am*. **97**, 209-218 (2004).

19. Song, F., Swinton, S. M., Difonzo, C. Neal, M. O. & Ragsdale, D. W. Profitability analysis of soybean aphid control treatments in three North-Central states. (Staff Paper No. 2006-24. Department of Agricultural Economics, Michigan State University, 2006).

20. Kim, K. S., Hill, C. B., Hartman, G. L., Mian, M. A. & Diers, B. W. Discovery of soybean aphid biotypes. *Crop Sci*. **48**, 923-928 (2008).

21. Krafsur, E. S., Obrycki, J. J. & Harwood, J. D. Comparative genetic studies of native and introduced Coccinellidae in North America. *Eur. J. Entomol.* **102**, 469-474 (2005).

22. Obrycki, J. J. & Tauber, M. J. Phenology of Three coccinellid species: thermal requirements for development. *Ann. Entomol. Soc. Am*. **74**, 31-36 (1981).

23. Xia, J. Y., Van der Werf, W. & Rabbinge, R. Temperature and prey density on bionomics of *Coccinella septempunctata* (Coleoptera: Coccinellidae) feeding on *Aphis gossypii* (Homoptera: Aphididae) on cotton. *Environ. Entomol*. **28**, 307-314 (1999).

24. Kumar, B. Temperature and photoperiod influence prey consumption and utilization by two sympatric *Coccinella* species (Coleoptera: Coccinellidae) in conspecific and heterospecific combinations. *Acta Entomol*. Sin. **58**, 297-307 (2015).

25. Ali, A. & Rizvi, P. Q. Effect of varying temperature on the survival and fecundity of *Coccinella septempunctata* (Coleoptera: Coccinellidae) fed on *Lipaphis erysimi*. *J. Entomol*. **5**, 133-137 (2008).

26. Katsoyannos, P., Stathas, G. J. & Kontodimas, D. C. Phenology of *Coccinella septempunctata* (Coleoptera: Coccinellidae) in central Greece. *BioControl*. **42**, 435-444 (1997).

Supplement 2. Stock establishment in this study.

This study used a commercial cultivar of soybeans (*G. max* cv. Kaohsiung No. 9), and collected soybean aphids and lady beetles from multiple sites to avoid idiosyncratic effects. The soybean cultivar was provided by the KDAIS and then cultivated from seeds, with a mixture of water, fertilizer (HYPONeX No.2, Hyponex Japan Co. Ltd.), and commercial soil (Kekkilä, Finland). Soybean aphids were collected from a soybean farm in Taipei (25°1′43.96″N, 121°34′42.65″E), a soybean farm in Tainan (23°12′12.34″N, 120° 6′0.69″E), and an experimental farm at the National Taiwan University (25°0′53.56″N, 121°32′26.81″E). Lady beetles were collected from the soybean farm in Tainan and another farm in Miaoli (24°29′41.56″N, 120°49′40.04″E). To establish the stock for experiments, collected soybean aphids and lady beetles were then raised on soybean plants in environmental growth chambers that simulate the average ambient condition of the soybean growth season in Taiwan (temperature = 24.5°C, relative humidity = 70 %, daylight = 12 hrs).

Supplement 3. The colonization experiment was conducted with open-top chambers (OTCs), each of which had one infested (central) soybean plant surrounded by four un-infested (neighbor) soybean plants, in the experimental farm of National Taiwan University.

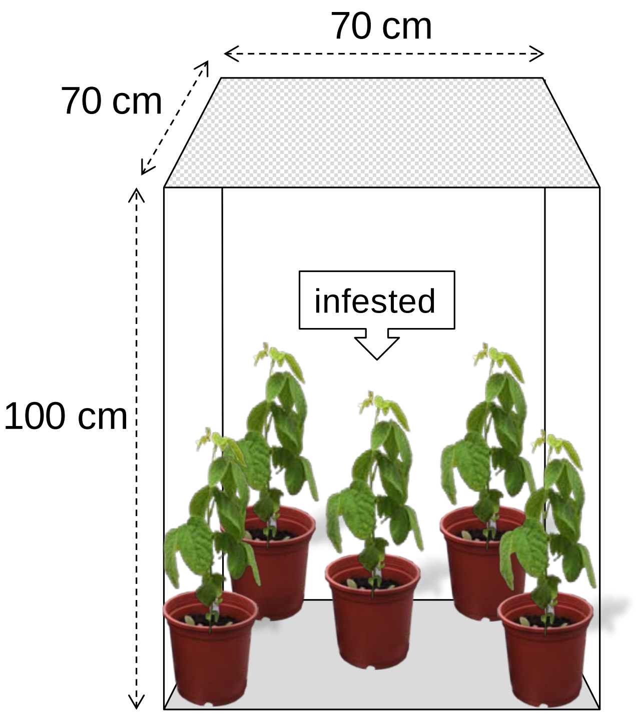

Each OTC (70 × 70 × 100 cm) was made of four transparent acrylic boards (70 x 100 x 0.5 cm), and its top was covered by fine silk meshes (400 holes/cm^2). The central plant was first infested at V1 stage with 10 apterous (wingless) aphids, which were allowed to reproduce apterous and alate aphids and then trimmed to 400 aphids, composed of 1% or 5% alate for the warming or ambient-temperature scenario, respectively. The central plant (around V2 stage) was then moved to an OTC and surrounded by four un-infested plants (V2

stage), with the distance between the infested (central) and un-infested plants at approximately 30 - 40 cm, comparable to the soybean spacing in real agricultural practice. One lady beetle was immediately added to an OTC that was randomly assigned for predator presence treatment. We counted alate and apterous aphids on both central and neighbor plants in each OTC every 24 hours in the first three days and every four days thereafter until the 23rd day. To ensure our predator treatment over time, we monitored OTCs twice a week and replaced a missing or dead lady beetle.

Each OTC was mainly used to cage plants and insects in the field, making our colonization experiment more realistic. Therefore, we did not monitor the temperature inside OTCs during the colonization experiment. To address whether the air temperature inside OTCs could be elevated, we deployed six temperature loggers (Spectrum WatchDog 1000 series 3684WD1) to measure the temperature inside and outside of three OTCs from December 6 to 9, 2016 in the same experimental plot. The result showed that the OTCs could increase daily mean air temperature by 0.87°C (20.89 inside vs. 20.02 outside; p-value = 0.02) (Table 1). Note that the degree of this warming varied hourly (Fig. 1).

Table 1. The daily mean air temperature inside and outside of OTCs (n = 3, 3)

	**Inside**	**Outside OTCs**
Daily mean temperature ± SD (°C)	20.89 ± 2.34	20.02 ± 1.33

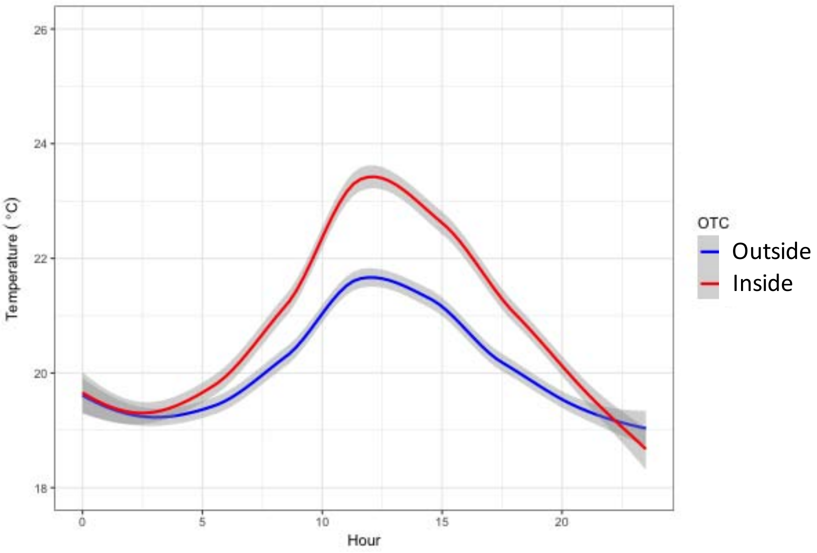

Figure 1. Hourly air temperature variations inside (red) or outside (blue) of OTCs. Gray area represents standard error.

159

Supplement 4. Temperature effect on alate aphid proportion. During the period when alates became common (proportion > 1%), aphid populations under 24.5°C (T1), 26.5°C (T2), and 28.5°C (T3) generated 5.51, 5.01, and 1.34% alate on average, respectively (Mean ± SE).

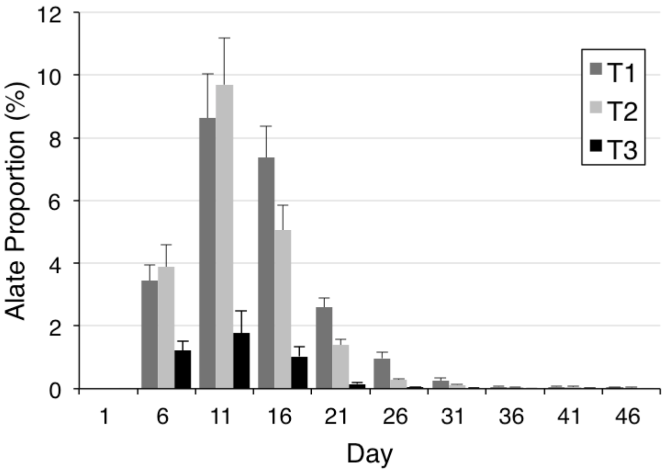

Supplement 5. Aphid population variables analyzed in this study

**Variable**	**Description**
Aphid population in an open-top chamber (OTC), including 1 central and 4 neighbor plants	
A/OTC	Aphid abundance in an OTC (alate + apterous)
AA/OTC	Alate aphid abundance in an OTC
Aphid population on the central plant in an open-top chamber	
A/C	Aphid abundance on the central plant (alate + apterous)
AA/C	Alate aphid abundance on the central plant
Aphid population on (4) neighbor plants in an open-top chamber	
A/N	Aphid abundance on neighbor plants (alate + apterous)
AA/N	Alate aphid abundance on neighbor plants

Supplement 6-1. Aphid abundance on the central soybean plant in an OTC under a combination of alate proportion treatment (A) and lady beetle treatment (L): A1 = low alate proportion (1%, warming scenario); A5 = high alate proportion (5%, ambient-temperature scenario); L0 = lady beetle absence; L1 = lady beetle presence. To facilitate the comparison of results, four sub-figures are listed: a) A1L0 vs. A1L1, b) A5L0 vs. A5L1, c) A1L0 vs. A5L0, and d) A1L1 vs. A5L1. Each point represents Mean ± SE. Statistical results for treatment effects are reported below the sub-figures (using GLS with AR1 to account for repeated measures of each aphid population).

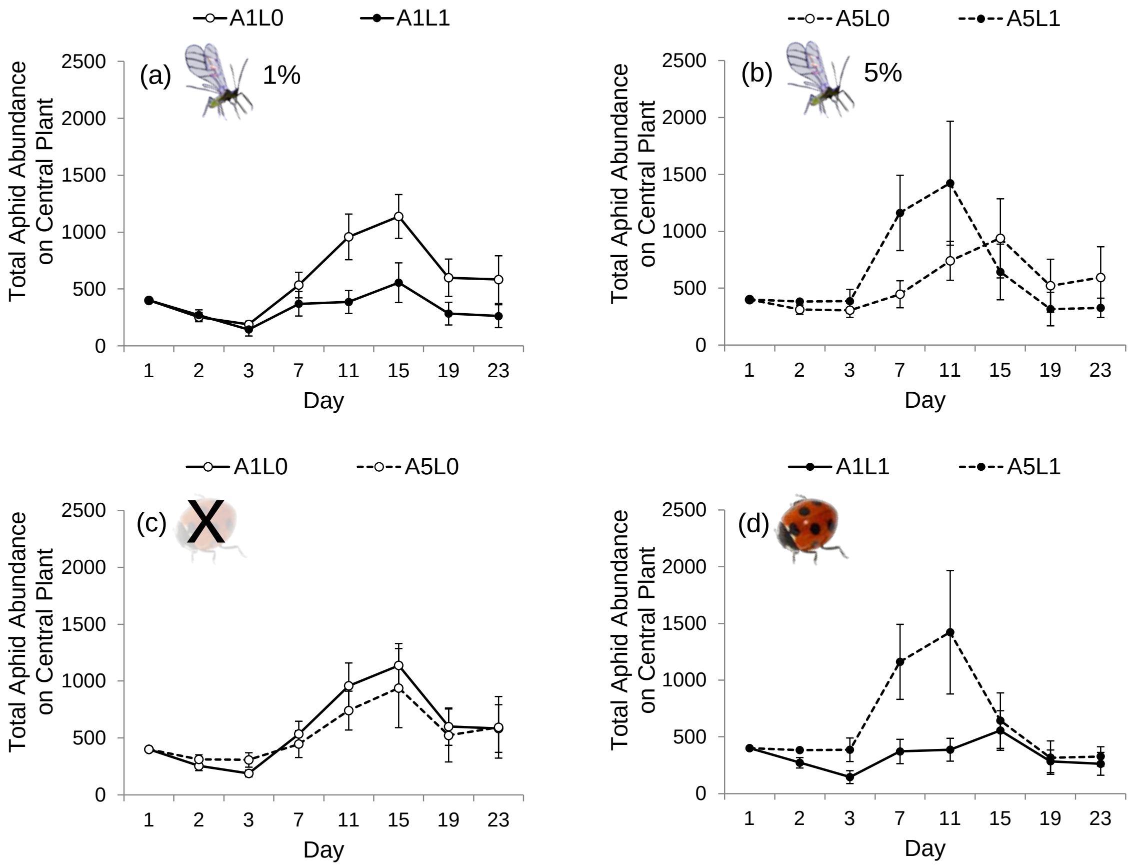

DenomDF = 152	Aphid Abundance on the Central Plant (A/C)		
Variable	NumDF	*F*	*P*
Alate proportion (A)	1	0.45	0.502
Lady beetle (L)	1	1.09	0.298
A × L	1	1.05	0.308
Time (in days) (D)	1	0.01	0.909
A × D	1	0.19	0.667
L × D	1	1.74	0.189
A × L × D	1	0.05	0.816

Supplement 6-2. Aphid abundance on neighbor soybean plants in an OTC under a combination of alate proportion treatment (A) and lady beetle treatment (L): A1 = low alate proportion (1%, warming scenario); A5 = high alate proportion (5%, ambient-temperature scenario); L0 = lady beetle absence; L1 = lady beetle presence. To facilitate the comparison of results, four sub-figures are listed: a) A1L0 vs. A1L1, b) A5L0 vs. A5L1, c) A1L0 vs. A5L0, and d) A1L1 vs. A5L1. Each point represents Mean ± SE. Statistical results for treatment effects are reported below the sub-figures (using GLS with AR1 to account for repeated measures of each aphid population).

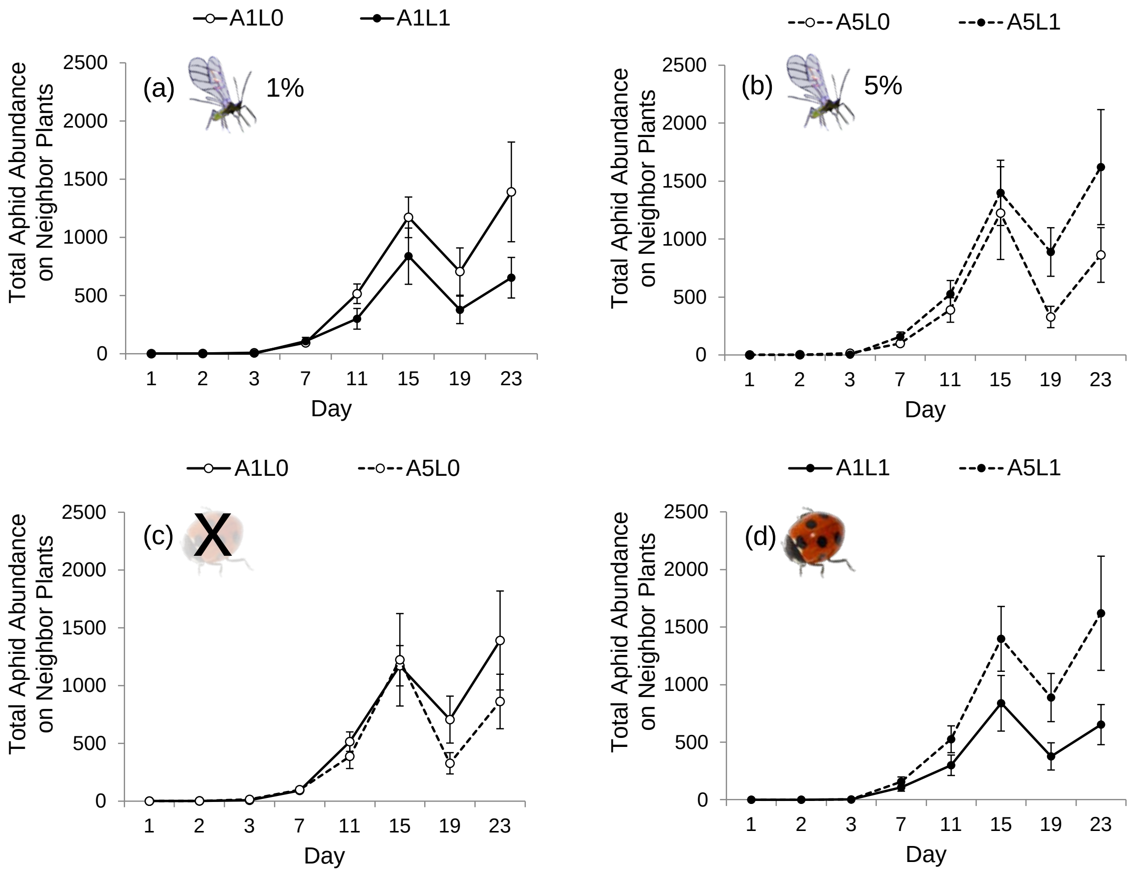

DenomDF = 152	Aphid Abundance on Neighbor Plants (A/N)		
Variable	NumDF	*F*	*P*
Alate proportion (A)	1	0.33	0.568
Lady beetle (L)	1	0.11	0.739
A × L	1	1.42	0.235
Time (in days) (D)	1	239.72	**<0.001**
A × D	1	0.06	0.804
L × D	1	0.36	0.551
A × L × D	1	1.78	0.184

Supplement 7-1. Alate aphid abundance on all soybean plants in an OTC under a combination of alate proportion treatment (A) and lady beetle treatment (L): A1 = low alate proportion (1%, warming scenario); A5 = high alate proportion (5%, ambient-temperature scenario); L0 = lady beetle absence; L1 = lady beetle presence. To facilitate the comparison of results, four sub-figures are listed: a) A1L0 vs. A1L1, b) A5L0 vs. A5L1, c) A1L0 vs. A5L0, and d) A1L1 vs. A5L1. Each point represents Mean ± SE. Statistical results for treatment effects are reported below the sub-figures (using GLS with AR1 to account for repeated measures of each aphid population).

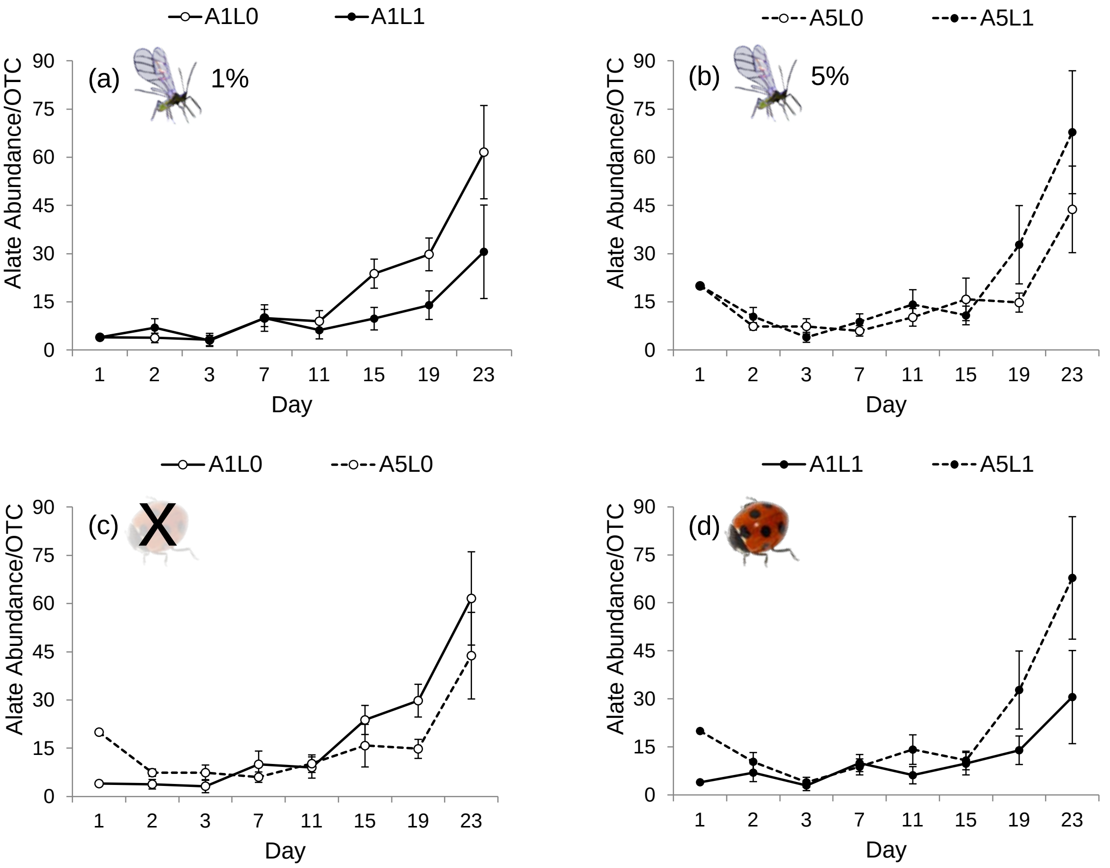

DenomDF = 152	Alate Aphid Abundance in an OTC (AA/OTC)		
Variable	NumDF	*F*	*P*
Alate proportion (A)	1	3.25	0.073
Lady beetle (L)	1	0.24	0.623
A × L	1	1.50	0.222
Time (in days) (D)	1	35.57	**<0.001**
A × D	1	1.97	0.162
L × D	1	0.48	0.489
A × L × D	1	4.28	**0.040**

Supplement 7-2. Alate aphid abundance on the central soybean plant in an OTC under a combination of alate proportion treatment (A) and lady beetle treatment (L): A1 = low alate proportion (1%, warming scenario); A5 = high alate proportion (5%, ambient-temperature scenario); L0 = lady beetle absence; L1 = lady beetle presence. To facilitate the comparison of results, four sub-figures are listed: a) A1L0 vs. A1L1, b) A5L0 vs. A5L1, c) A1L0 vs. A5L0, and d) A1L1 vs. A5L1. Each point represents Mean ± SE. Statistical results for treatment effects are reported below the sub-figures (using GLS with AR1 to account for repeated measures of each aphid population).

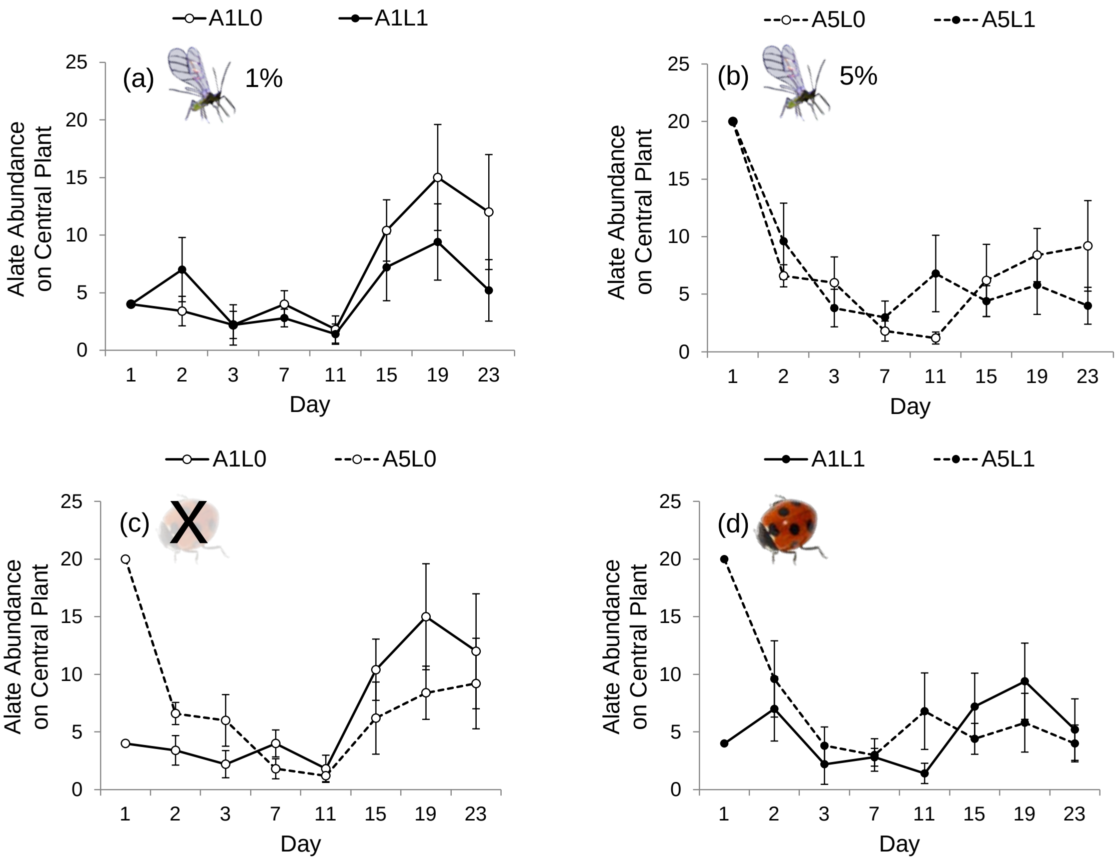

DenomDF = 152	Alate Aphid Abundance on the Central Plant (AA/C)		
Variable	NumDF	*F*	*P*
Alate proportion (A)	1	0.50	0.480
Lady beetle (L)	1	0.26	0.608
A × L	1	2.72	0.101
Time (in days) (D)	1	144.07	**<0.001**
A × D	1	0.19	0.665
L × D	1	0.02	0.880
A × L × D	1	6.81	**0.010**

Supplement 7-3. Alate aphid abundance on four neighbor soybean plants in an OTC under a combination of alate proportion treatment (A) and lady beetle treatment (L): A1 = low alate proportion (1%, warming scenario); A5 = high alate proportion (5%, ambient-temperature scenario); L0 = lady beetle absence; L1 = lady beetle presence. To facilitate the comparison of results, four sub-figures are listed: a) A1L0 vs. A1L1, b) A5L0 vs. A5L1, c) A1L0 vs. A5L0, and d) A1L1 vs. A5L1. Each point represents Mean ± SE. Statistical results for treatment effects are reported below the sub-figures (using GLS with AR1 to account for repeated measures of each aphid population).

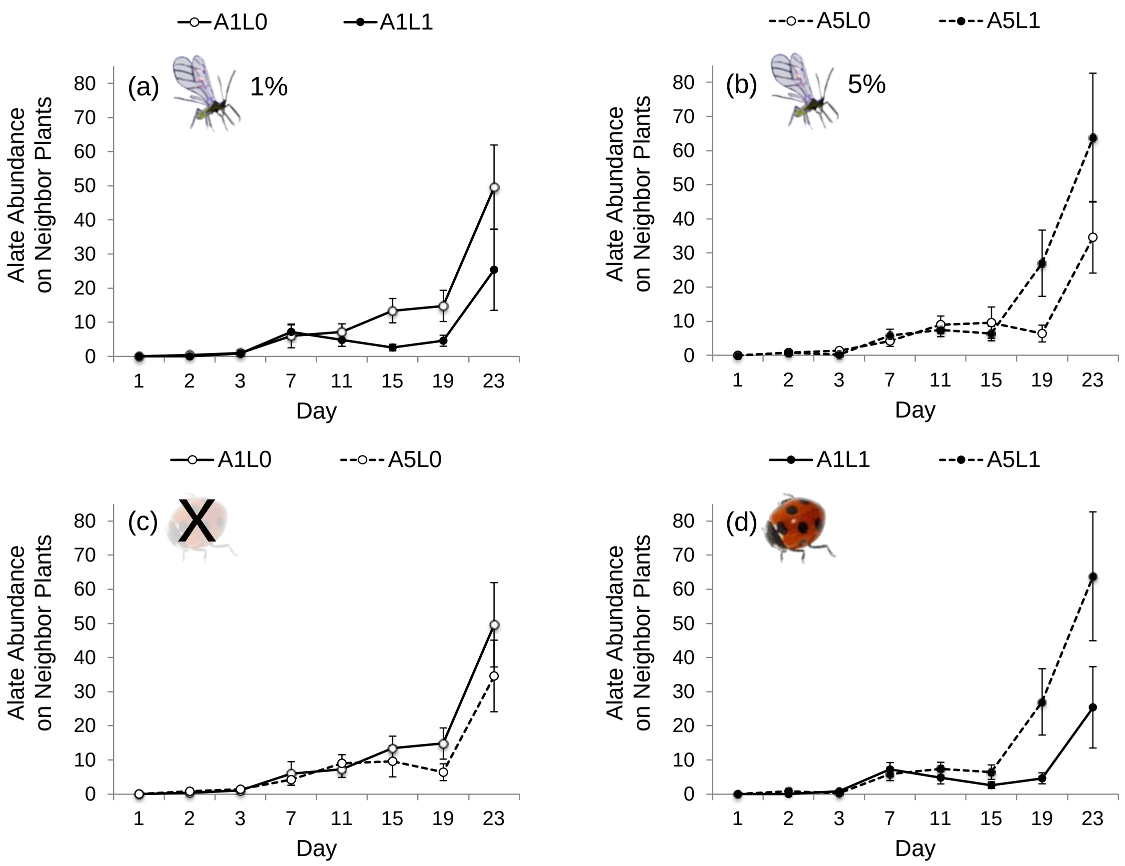

DenomDF = 152	Alate Aphid Abundance on Neighbor Plants (AA/N)		
Variable	NumDF	*F*	*P*
Alate proportion (A)	1	0.47	0.495
Lady beetle (L)	1	0.32	0.575
A × L	1	1.79	0.183
Time (in days) (D)	1	140.85	**<0.001**
A × D	1	0.02	0.891
L × D	1	0.01	0.930
A × L × D	1	5.64	**0.019**

Supplement 8. The thermal performance curve of soybean aphids (redrawn from McCornack et al. 2004). The performance plateau around the optimum temperature range likely explains the lack of warming impact on aphid population size in this study (24.5 and 28.5°C represent ambient and 4°C warming treatments, respectively).

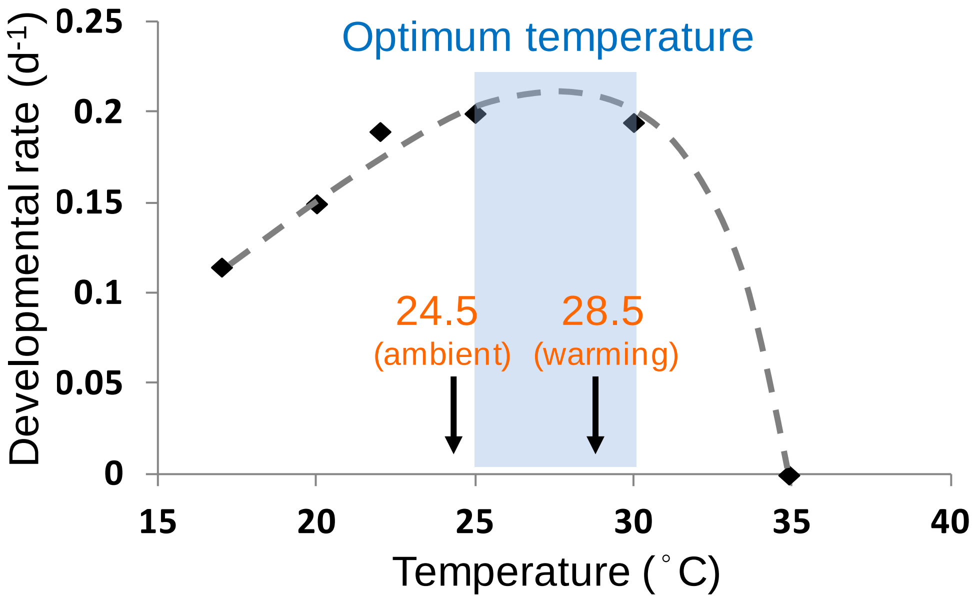

Supplement 9. A population dynamics model to study alate and lady beetle effects on aphid colonization.

The colonization experiment revealed an interaction between alate proportion and lady beetle treatments: lady beetle presence inhibited and could facilitate aphid colonization under the lower and higher alate proportion treatments (1% vs. 5%), respectively. To help understand the underlying mechanisms for aphid population dynamics, we developed a five-patch population dynamics model based on literature and our experimental data (see below for detailed model descriptions). The model would generate similar results to those in the colonization experiment (i.e., an interaction between alate proportion and lady beetle treatments), when the following conditions were met: 1) increasing alate proportion reduced lady beetle predation on aphids, and 2) lady beetle presence enhanced the intrinsic growth rate of aphids on the central plant. In short, the alate proportion-lady beetle interaction observed in the colonization experiment could be supported by our population dynamics model.

In the model, aphids moves from an initially colonized patch on the central plant (N_0) to four neighboring patches (plants) (N_i):

$$\frac{dN_0}{dt} = x_r r N_0\left(1-\frac{N_0}{K}\right) - x_L L \frac{N_0^{\,2}}{(h+N_0)\sum_{j=0}^{4} N_j} - d\,\left(N_0\right)^2 \qquad \text{(D1a)}$$

$$\frac{dN_i}{dt} = rN_i\left(1 - \frac{N_i}{K}\right) - x_L L \frac{N_i^{\,2}}{(h+N_i)\sum_{j=0}^{4} N_j} - d\,\left(N_i\right)^2 + \frac{pd\left(N_0\right)^2}{4} \qquad (i = 1 \text{ to } 4)\ \text{(D1b)}$$

The aphid populations have logistic growth, with intrinsic growth rate r and carrying capacity K. The parameter L denotes daily predation capacity per lady beetle, which is allocated to each patch (foraging ground) depending on the relative aphid abundance[1,2]. In each patch, the lady beetle (predator) is assumed to have a Holling type-II with half saturation constant h. Because aphid population performance may depend on alate proportion and/or lady beetle presence, two parameters are introduced: x_r is the lady beetle effect on aphids' population growth rate, and x_L is the alate effect on a lady beetle's predation efficiency. N_j refers to the aphid population on the central or neighbor plants. The parameter d determines the density-dependent emigration, assuming that crowding enhances aphid dispersal[3,4], and p discounts immigration rate into neighbor patches due to dispersal failure (i.e., $p < 1$). To mimic the initial condition in our colonization experiment, the model assumes that aphids always disperse from the central to neighbor patches.

Using this model, we examined whether the following two hypothesized mechanisms can lead to the interaction effect between alate proportion and lady beetle treatments observed in the colonization experiment: 1) increasing alate proportion reduces lady beetle predation on aphids (i.e. $x_L < 1$), and 2) lady beetle presence enhances the intrinsic growth

rate of aphids on the central plant (i.e. $x_r > 1$) (see references in the Discussion section). To do so, we manipulated the coefficients x_L and x_r and examined their effects on aphid population dynamics. Based on the literature and our experimental data, other parameter values were set as follows: $r = 0.3$, $K = 2000$, $L = 70$ (in day^{-1}), $h = 140$, $x_d = 0.0001$, $d = 0.07$[5,6,7,8]. The initial population sizes were $N_0(0) = 400$ and $N_i(0) = 0$ ($i = 1$ to 4). All simulations were done by wxMaxima v. 15.04.0.

We found that the simulated population dynamics were similar to those observed in the colonization experiment when 1) x_L was sufficiently small ($x_L < 0.5$) and 2) x_r was sufficiently large ($x_r > 1.2$). Specifically, lady beetle presence enhanced and depressed aphid population growth in 5% and 1% alate condition, respectively (Fig. a for total aphid abundance). However, when either assumption was violated, the model failed to generate the pattern, regardless of the value of the other parameter (Fig. b, c, d, e). These simulation results generally support the hypothesized mechanisms underlying the interaction effect between alate proportion and lady beetle treatments, as well as reveal the condition under which the mechanisms will work. To fully confirm this idea, experimental estimates of the parameters x_L and x_r will be necessary.

Figure a-f. The simulated aphid population dynamics: (x_L, x_r) = (1/7, 4/3) (a), (1, 4/3) (b), (1, 2) (c), (1/7, 1) (d), (1/70, 1) (e), and (1, 1) (f). In each panel, dashed lines with different symbols denote four experimental treatments (alate proportion x lady beetle treatment): red cross (1% alate without lady beetle), blue cross (5% alate without lady beetle), red dot (1% alate with lady beetle), and blue dot (5% alate with lady beetle). (x_L, x_r) = (1, 1) corresponds to the absence of the hypothesized mechanisms.

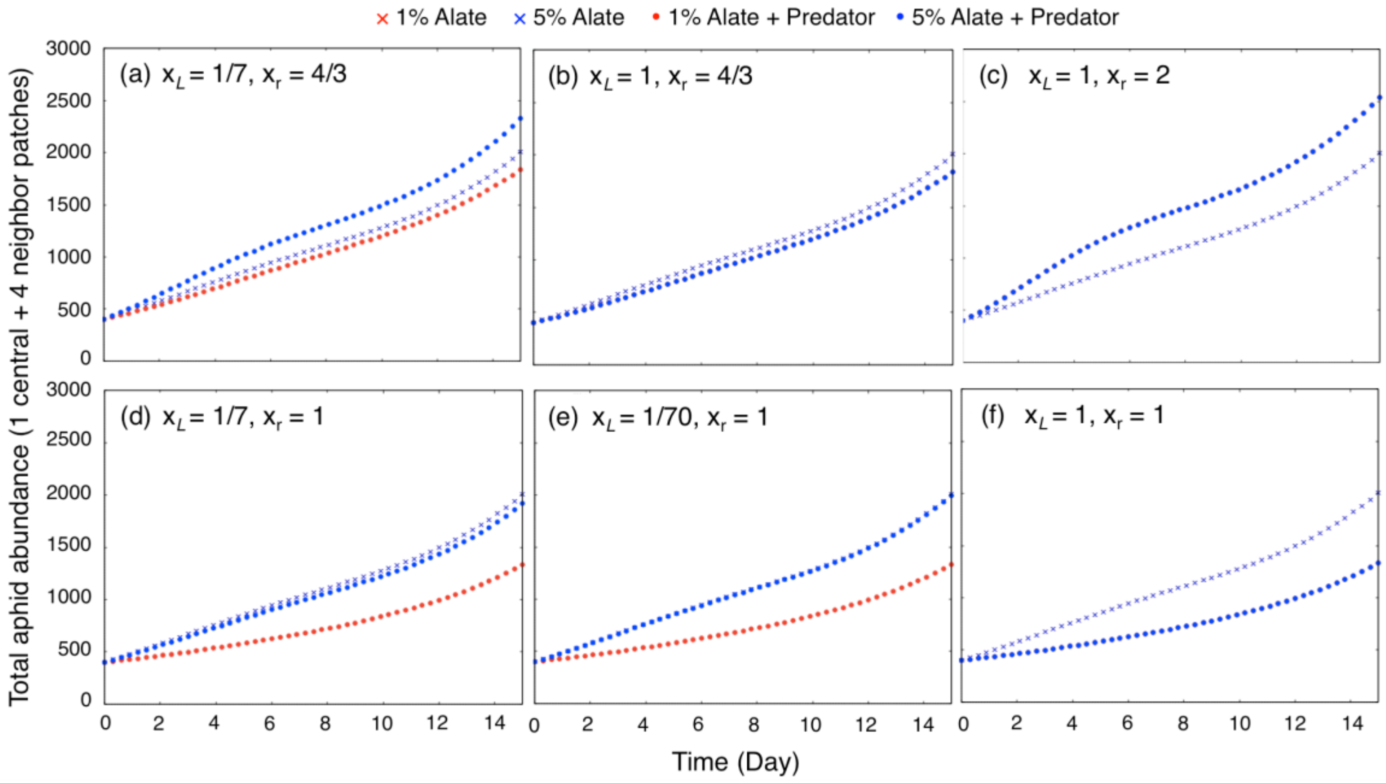

Literature Cited for Supplement 9

1. Werf, W., Evans, E. W. & Powell, J. Measuring and modelling the dispersal of *Coccinella septempunctata* (Coleoptera: Coccinellidae) in alfalfa fields. *Eur. J. Entomol.*

97, 487 (2000).

2. Evans, E. W. Searching and reproductive behaviour of female aphidophagous ladybirds (Coleoptera: Coccinellidae): a review. *Eur J Entomol*. **100**, 1-10 (2003).
3. Hodgson, C. Dispersal of apterous aphids (Homoptera: Aphididae) from their host plant and its significance. *Bull. Entomol. Res*. **81**, 417-427 (1991).
4. Karley, A. J., Parker, W. E., Pitchford, J. W. & Douglas, A. E. The mid-season crash in aphid populations: why and how does it occur? *Ecol. Entomol*. **29**, 383-388 (2004).
5. Suhail, A., Sabir, A. M., Hussain, A. & Saeed, A. Predatory efficacy of Coccinella septempunctata L. on Cotton Aphids, *Aphis gossypii* Glov. *Pak. J. of Bio. Sci*. **2**, 603-605 (1999).
6. McCornack, B. P., Ragsdale, D. W. & Venette, R. C. Demography of soybean aphid (Homoptera: Aphididae) at summer temperatures. *J. Econ. Entomol*. **97**, 854-861 (2004).
7. Costamagna, A. C., Van der Werf, W., Bianchi, F. J. J. A. & Landis, D. A. An exponential growth model with decreasing r captures bottom-up effects on the population growth of *Aphis glycines* Matsumura (Hemiptera: Aphididae). *Agric. Forest Entomol*. **9**, 297-305 (2007).
8. Kummel, M., Brown, D. & Bruder, A. How the aphids got their spots: predation drives self-organization of aphid colonies in a patchy habitat. *Oikos*. **122**, 896-906 (2013).

Supplement 10. Potential caveats of this study

This study used both warming and colonization experiments to clarity the mechanisms underlying warming effects. Considering logistics, the warming experiment was conducted under constant temperature on the basis of the average temperature of the soybean growth season. In future studies, the temperature should be fluctuated at least hourly to mimic natural dynamics, which might increase or reduce insect performance[1]. Another caveat of this study is that the field colonization experiment involved applying the warming experiment results (i.e., lower alate proportion under warming) in simulating the warming impact on aphid colonization, instead of applying real temperature manipulation. Although this approach may overlook the potential warming impact on plants and lady beetles, the conclusions of the colonization experiment may not be affected on the basis of bottom-up and top-down perspectives. First, warming did not affect the leaf C/N ratio in the warming experiment ($F = 1.06$, $P = 0.38$) or leaf secondary compounds in a previous study (Lin and Ho, *unpublished data*), suggesting that the inclusion of warming treatment may not have altered plant quality (bottom-up control of aphids) in the colonization experiment. Second, modest warming may increase lady beetles' developmental rate, predation efficiency, and top-down control of aphids[2, 3, but 4] (Lin and Ho *unpublished data*), suggesting that the

inclusion of warming treatment in the colonization experiment may strengthen our conclusion that warming-induced effect (i.e., alate reduction) has a potential to increase the top-down control (biocontrol) of aphids by lady beetles (Figure below).

Figure. An inclusion of warming treatment in the colonization experiment may strengthen the conclusion of this study because 1) warming should increase the searching and predation rate of our lady beetle species in our focal temperature range (i.e., a 2-4°C warming above 24.5°C (ambient))[2], and 2) this stronger predation pressure should impede aphid population growth (colonization). The hypothesized result (with warming treatment) and the reported result (without warming treatment) for the colonization experiment are illustrated in dash and solid red lines, respectively.

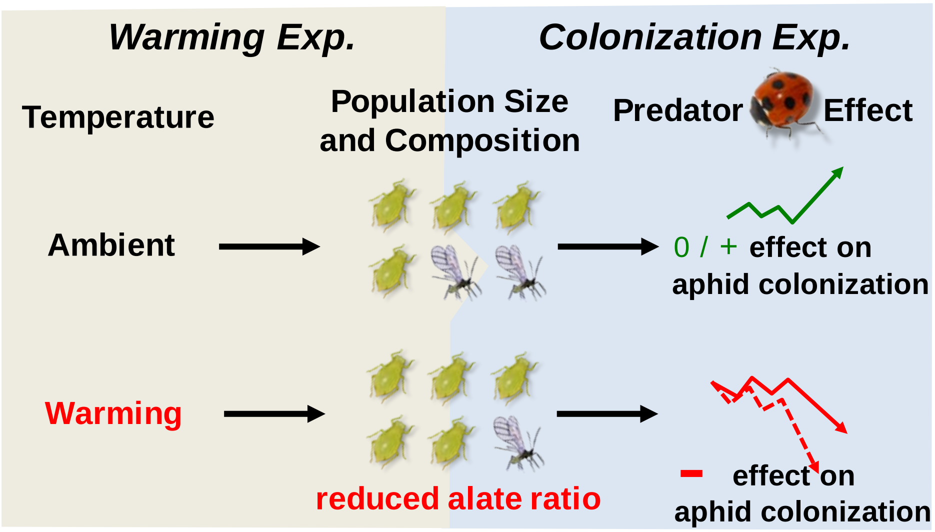

Literature Cited for Supplement 10

1. Colinet, H., Sinclair, B. J., Vernon, P. & Renault, D. Insects in fluctuating thermal environments. *Annu. Rev. Entomol.* **60**, 123-140 (2015).
2. Xia, J. Y., R. Rabbinge & W. Van Der Werf. Multistage functional responses in a ladybeetle-aphid system: scaling up from the laboratory to the field. *Environmental Entomology* **32**: p151-162 (2003).
3. Katsarou, I., Margaritopoulos, J. T., Tsitsipis, J. A., Perdikis, D. C. & Zarpas, K. D. Effect of temperature on development, growth and feeding of *Coccinella septempunctata* and *Hippodamia convergens* reared on the tobacco aphid, *Myzus persicae nicotianae*. *BioControl*. **50**, 565-588 (2005).
4. Harmon, J. P., N. A. Moran & A. R. Ives. Species response to environmental change: impacts of food web interactions and evolution. *Science* **323** (5919): 1347-1350 (2009).
